# Protocol for the development of a core outcome set for evaluating mixed-diagnosis falls prevention interventions for people with Multiple Sclerosis, Parkinson’s Disease and stroke

**DOI:** 10.12688/hrbopenres.13459.1

**Published:** 2021-11-26

**Authors:** Nicola O'Malley, Susan Coote, Amanda M Clifford

**Affiliations:** 1 School of Allied Health, Faculty of Education and Health Sciences, University of Limerick, Limerick, Ireland; 2 Ageing Research Centre, Health Research Institute, University of Limerick, Limerick, Ireland; 3 Centre of Physical Activity for Health, Health Research Institute, University of Limerick, Limerick, Ireland; 4 Multiple Sclerosis Society of Ireland, Limerick, Ireland

**Keywords:** Consensus methods, Core outcome set, Falls, Parkinson’s Disease, Multiple Sclerosis, Stroke

## Abstract

**Background:** Given the high incidence of falls and their associated negative effects, the development of effective falls prevention interventions for people with Multiple Sclerosis (MS), Parkinson's Disease (PD) and stroke is a priority. Currently the implementation of condition-specific falls prevention interventions is challenging in the community due to lack of participants and resources. Given the similarities in falls risk factors across stroke, PD and MS, the design of mixed-diagnosis interventions for groups comprising of people with these three neurological conditions may solve these implementation challenges. Having a core outcome set (COS) for evaluating these interventions would enable the comparison and combination of data, thereby facilitating progress in this research area. Therefore, the aim of this research study is to develop a COS for evaluating mixed-diagnosis falls prevention interventions for people with MS, PD and stroke.

**Methods:** This will be a mixed-methods, international, multi-perspective Delphi consensus study with five stages. Stage one will involve the identification of potential outcomes through a systematic literature search, patient focus groups, and consultation with our Public and Patient Involvement (PPI) panel. The second stage will be the development of the Delphi survey using the outcomes elicited from stage one. Stage three will be the prioritisation of outcomes using a two-round online Delphi survey involving patients, clinicians, researchers and policy-makers/service-planners. The fourth stage will be to identify and recommend outcome measures and definitions. The final stage will be a consensus meeting with representatives from each stakeholder group to agree upon the final COS.

**Discussion: **Adoption of this COS in future trials investigating the effectiveness of mixed-diagnosis falls prevention interventions for people with MS, PD and stroke will facilitate the comparison and combination of research findings. This should translate into improved decision-making by service-planners/policy-makers and clinicians regarding the implementation of evidence-based falls prevention interventions into practice.

## Introduction

Neurological conditions are the main cause of disability globally and, therefore, people with neurological conditions have high rehabilitation needs ^1, 2^. In Ireland, three common neurological conditions with high falls rates are Multiple Sclerosis (MS), Parkinson’s Disease (PD) and stroke and, consequently, there is a need to develop effective falls prevention interventions to facilitate evidence-based service planning and resource allocation ^3^. More than 50% of people with MS and PD fall in a three-month or six-month period, respectively, while as many as 73% of people will experience a fall in their first 12 months post-stroke ^4– 6^. Falls have a number of physical and psychosocial effects on individuals with these neurological conditions including physical injury, fear of falling, activity curtailment, reduced independence and decreased quality of life ^7– 13^. In addition, the consequences of falls increase strain on healthcare systems, due to higher acute healthcare service needs, and greater requirement for home-care and/or institutional-care ^8– 10, 14^. As a result of the high incidence of falls and the associated negative consequences, falls prevention for people with MS, PD and stroke is an important topic for research and the provision of healthcare services.

In recent years, there has been a proliferation in condition-specific falls prevention research among people with MS, PD and stroke. However, the implementation of these single-diagnosis falls prevention interventions is proving challenging in the community and primary care due to insufficient numbers of participants and resources to run separate group-based programmes ^15^. While there are differences in the underlying pathophysiology of these three neurological conditions ^16– 19^, research has identified many common physiological, psychosocial, environmental and behavioural falls risk factors across the three conditions ^20– 29^. Given these similarities in falls risk factors across stroke, PD and MS, the development of mixed-diagnosis multifactorial interventions for these three neurological conditions, with the scope to tailor elements such as education and exercise to the individuals’ needs, is a practical solution to bridge the intervention gap, resolving these barriers to implementation and the provision of services in the community.

Progress in the development and evaluation of single-diagnosis interventions designed to reduce falls thus far has been hampered by substantial variation in the outcomes assessed across studies. The heterogeneity in outcomes and/or how they are measured is repeatedly acknowledged as a limitation as it inhibits the synthesis and cross-comparison of evidence, thereby preventing researchers and clinicians from evaluating and interpreting the effectiveness of falls prevention interventions ^30– 33^. The heterogeneity in outcomes assessed across studies is reflective of the current absence of a gold standard method to evaluate falls prevention interventions among people with these neurological conditions. The development of a core outcome set (COS) for evaluating falls prevention interventions among mixed-diagnosis groups comprising of people with MS, PD and stroke would mean that similar pitfalls in falls research among mixed-diagnosis groups could be avoided.

A core outcome set (COS) is a standardised set of outcomes that should be assessed and reported at a minimum in all trials pertaining to a specific health construct, condition or population ^34^. When developing a COS, it is first necessary to gain consensus regarding ‘what’ to measure. When this has been completed, the second step is to determine ‘how’ to define and assess the outcomes that have been selected ^34^. Having a COS for evaluating mixed-diagnosis falls prevention interventions among adults with MS, PD and stroke will enable the comparison and combination of data, thereby ensuring that research findings are relevant, useful and useable ^35^. Consequently, the aim of this study is to develop and disseminate a COS for evaluating mixed-diagnosis falls prevention interventions for people with MS, PD and stroke.

The following are the objectives of this study:

1. To identify all potential outcomes for mixed-diagnosis falls prevention interventions for people with MS, PD and stroke through a review of the literature and focus groups with people living with these neurological conditions.

2. To achieve consensus on a COS for evaluating mixed-diagnosis falls prevention interventions for people with MS, PD and stroke using the Delphi technique and a consensus meeting.

## Methods

### Protocol and prospective registration

This study was prospectively registered with the Core Outcome Measures in Effectiveness Trials (COMET) Initiative on the 24th September 2021 and is available online ( https://www.comet-initiative.org/Studies/Details/1940). This protocol was developed and reported in adherence with the Core Outcome Set-STAndardised Protocol (COS-STAP) Items ^36, 37^.

### Scope

This COS, and the corresponding definitions and outcome measures, should apply to both clinical practice and all research where the aim is to evaluate falls prevention interventions for mixed-diagnosis groups comprising of people with MS, PD or stroke. The target population for this COS is adults (≥18 years) with MS, PD and stroke, according to a confirmed diagnostic criterion, with the ability to mobilise and stand independently (with or without the use of an aid), of any gender and disease duration. This outcome set should be applied to interventions where the aim is to reduce falls among the target population.

### Participants

A purposive and iterative approach will be used to identify individuals to participate in the international Delphi survey. Survey respondents will consist of individuals from each of the following key stakeholder groups: researchers, clinicians, people living with MS, PD and stroke, and service-planners/policy-makers. While feedback between rounds will be generated based on stakeholder group, only outcomes that reach consensus for inclusion based on the combined scoring of all stakeholder groups will be included in the final COS. Therefore, to ensure that the final COS is reflective of the opinions of all relevant stakeholder groups and is not influenced by the relative proportion of stakeholders participating, we will aim to recruit a similar number of participants from each stakeholder group ^38^. There is currently an absence of robust methods to calculate the required sample size for a Delphi survey with the aim of achieving consensus on a COS, however, it is generally accepted that the more participants representing each stakeholder group, the greater the reliability and generalisability of the COS ^38, 39^. It has been suggested that at a minimum a panel would consist of 10 to 18 participants per stakeholder group ^40^. Consequently, we will aim to recruit approximately 15 individuals from each stakeholder group in case of attrition between rounds. Every effort will also be made to achieve a gender balance in the participants and to recruit participants from a wide geographic distribution.

Researchers, clinicians and policy-makers/service-planners will be recruited via their email address, which will be identified from research articles and reviews, professional body email lists, Twitter and special interest groups. Patient participants will be recruited through support groups/community services for people with PD, MS and stroke. Social media and other communications of relevant organisations will also be used. Potential participants will be provided with an information leaflet outlining the rationale, objectives and methods for the consensus, and invited to participate. The research team will follow-up with those who express interest in the study through phone call or email to address any questions that the individual may have regarding the study. Recruitment will adhere to principles of purposeful and snowball sampling. Eligibility criteria are as follows: adults (aged 18 years or over) who are able to read and write in English and are (a) living with a confirmed diagnosis of MS, PD and/or stroke; (b) researchers actively involved in falls prevention research for people with these neurological conditions and have a minimum of three peer-reviewed publications in this research field; (c) clinicians currently providing interventions to individuals with these neurological conditions; or (d) service-planners/policy-makers involved in decision-making regarding the provision of falls prevention services.

The retention of participants in Delphi surveys has proven challenging at times for COS developers. Failure to retain participants in this study has the potential to introduce attrition bias if those who do not continue to participate have differing viewpoints to those who complete all rounds of the survey ^38^. Attrition bias will be assessed at each round by comparing the average score for each outcome of those who respond to the survey to those who do not, identifying any substantial differences in scoring ^38^. Steps will be taken throughout the study process to maximise retention including personalised reminders, public involvement in the development of surveys to ensure the language is appropriate and understandable, and a short wait between rounds ^38, 41^.

### Design

**Figure 1.**
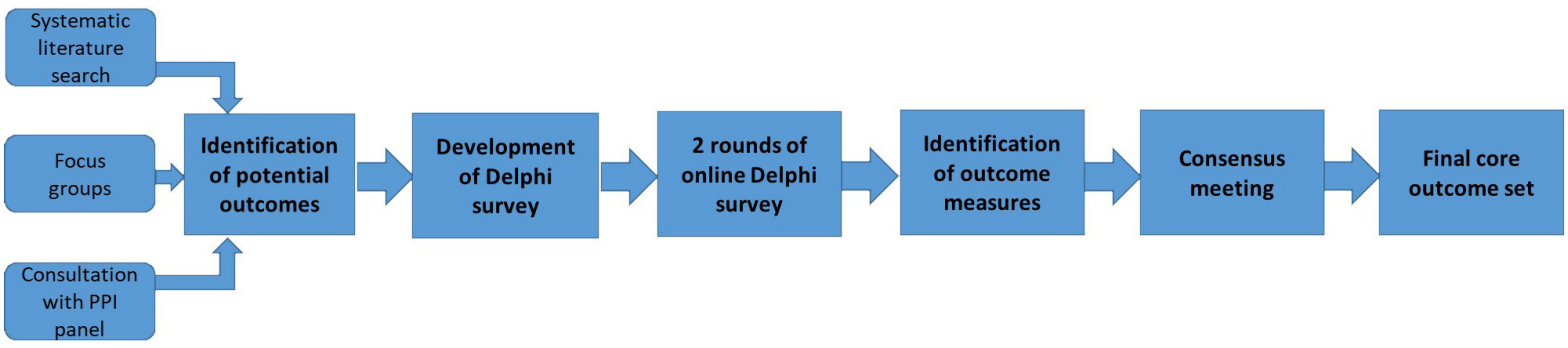
Flowchart of study design.

This will be an international, multi-perspective consensus study, which will involve five stages as demonstrated in Figure 1:

1. Identification of potential outcomes through a systematic literature search, patient focus groups and consultation with our Public and Patient Involvement (PPI) panel.

2. Development of the Delphi survey.

3. Prioritisation of outcomes using an electronic Delphi survey.

4. Identification and standardisation of outcome definitions and measures.

5. Agreement on the final COS at a consensus meeting.

### Stage 1: identifying potential outcomes

***Systematic search of the literature.*** We performed an umbrella review of systematic reviews investigating the effectiveness of falls prevention interventions for people with MS, PD and stroke ^42^. This umbrella review was registered with PROSPERO (CRD42020175409) and the protocol published in an open access repository ^43^. A systematic literature search was conducted using 15 electronic databases, grey literature searches and hand-screening of reference lists. Systematic reviews of randomised-controlled trials and non-randomised studies of intervention investigating the effectiveness of non-pharmacological and non-surgical interventions on falls among people with MS, PD and stroke were included. A total of 18 systematic reviews met the predefined inclusion criteria, representing 73 unique primary studies. The reported outcomes, how they were defined, the outcome measures used and time points for measurement were extracted from each systematic review. In instances where these were not reported or the details were unclear in the systematic review, the authors retrieved the original primary studies to extract this data. The outputs from this umbrella review will be used to generate the initial outcome list. All identified outcomes will be presented for rating in the Delphi survey.

***Focus groups with people living with MS, PD and stroke.*** Outcomes collated through a review of the literature are primarily reflective of outcomes that are deemed important by researchers, potentially overlooking outcomes that are meaningful to patients ^44^. Consequently, some COS developers have begun undertaking qualitative studies with patients and/or other stakeholders to ensure that a comprehensive list of outcomes, including those that are important and meaningful to all stakeholders, are considered for inclusion in the COS ^38, 44, 45^. This study will employ a qualitative design, using focus groups to explore what outcomes for evaluating falls prevention interventions are important to people living with MS, PD and stroke. Participants will include individuals aged 18 years and over who self-identify as having a confirmed diagnosis of MS, PD and/or stroke. In light of the guidance from the World Health Organisation and Health Service Executive regarding physical distancing, these focus groups will take place using an online teleconferencing platform. A semi-structured topic guide consisting of open-ended questions will be used by the facilitator to direct the focus group. Focus groups will be audio-recorded, transcribed and analysed using thematic analysis. From this analysis, a list of outcomes will be generated. The data generated from this qualitative study will also be used to provide context around why the outcomes discussed are important to patients and to develop lay definitions for outcomes presented in the Delphi survey.

***Consultation with PPI panel.*** The lists of outcomes generated from the literature review and focus groups will be reviewed and discussed by the research team and PPI panel to identify all distinct outcomes to be included in the Delphi survey. Given their expertise and insights into practice evidence, patient evidence and contextual factors, three key aspects of evidence-based practice and treatment decision-making ^46, 47^, our PPI panel will also have the opportunity to suggest additional outcomes that they think are potentially important/meaningful but are not included in the list.

### Stage 2: development of the Delphi survey

The Delphi method has four fundamental features: sequential questionnaires, anonymity of participant responses, the provision of controlled feedback between questionnaire rounds, and the aggregation of participant responses to determine if and when consensus has been achieved ^48^. The controlled Delphi method is favoured over less structured methods used to gain consensus, such as round-table discussions, as there is no direct contact or interactions between participants, thereby reducing the likelihood of responses being influenced by domineering individuals ^48, 49^.

A sequential two-round electronic, international Delphi survey will be completed involving key stakeholders to develop a preliminary COS. The online software Qualtrics (Provo, UT) will be used to administer the survey. Outcomes identified in stage one will be listed in alphabetical order in the survey to avoid potential weighting ^50^. This survey will be developed with input from our PPI panel to ensure ease of completion and clarity. Following its development, the survey will be piloted and will be modified as required prior to formal circulation to participants. Each round of the survey will remain open for two weeks, with a reminder email sent out to participants three working days before closure. The data obtained from each round will be analysed and presented to the participants in the next round. It is proposed that the prioritisation of outcomes will comprise of two rounds, however, the determination of the number of rounds will be a dynamic process with additional or less rounds included as appropriate ^38^.

Delphi survey participants will be asked to score individual outcomes using the Grading of Recommendations Assessment, Development and Evaluations (GRADE) nine-point Likert scale, with 1–3 signifying an outcome of limited importance, 4–6 an important but not critical outcome, and 7–9 indicating a critically important outcome ^34, 51^. The ‘70/15%’ consensus definition will be used to determine whether consensus has been achieved ^34^. Consensus that an outcome should be included in the final COS will be defined as 70% or greater of the participants scoring it as critically important (7–9) and less than 15% scoring it as having limited importance (1–3) ^34^. Consensus regarding whether an outcome should be excluded from the COS will be defined as 70% or greater of the respondents scoring it as having limited importance (1–3) and less than 15% scoring it as critically important (7–9) ^34^. Score distributions outside of those outlined above will signify a lack of agreement with respect to the inclusion of an outcome in the COS ^34^.

### Stage 3: prioritisation of outcomes

***Delphi survey – round one.*** During round one, participants will provide their demographic data including gender, age, nationality, stakeholder group, profession and years of experience. Respondents will be each provided with a unique identifier to facilitate future anonymity. Participants will be asked to rank each outcome using the nine-point Likert scale described above. Participants will also be encouraged to give the rationale for their scores (each item in the survey will have a comment box). These responses will be summarised using content analysis and these data will be provided to participants in the next round to provide context to the scores given to outcomes. Finally, participants will have the option to suggest additional outcomes for inclusion in the next round of the survey. Additional outcomes suggested in this round will be reviewed by two members of the research team to determine if they represent new outcomes ^50^. All outcomes will be brought forward from round one to round two.

***Delphi survey – round two.*** Individuals who participated in round one of the survey will be provided with the descriptive statistics of their own and other respondents’ scores from round one, in addition to a summary of the reasons that individuals gave for their scoring of each outcome. Descriptive statistics will be calculated for the panel as a whole and for each stakeholder group, with all participants being provided both sets of statistics. Participants will be asked to reflect on these summaries and statistics provided for each stakeholder group and their own scores before being asked to rescore all outcomes from round one and to score any new outcomes suggested by participants using the nine-point scoring system. If participants change their score for an outcome in round two, they will be encouraged to provide their rationale for this. Following round two of the survey, outcomes will be divided into three categories: category A (those meeting the criteria for consensus on inclusion – high agreement and high support), category B (those not achieving consensus - low agreement and mixed support) or category C (those meeting criteria for consensus on exclusion - high agreement and low support) ^52^. Category A outcomes will be added to the preliminary COS. Category B outcomes will be added to a list called ‘supplementary outcomes’. Category C outcomes will not be involved in any further discussions and will not be considered for inclusion in the final COS. At the end of round two of the survey, there will be a question included asking respondents if they would be interested in taking part in the virtual face-to-face consensus meeting.

### Stage 4: identification and standardisation of outcome definitions and measures

The COnsensus-based Standards for the selection of health status Measurement Instruments (COSMIN) recommends a thorough methodology for in-depth evaluation and selection of outcome instruments ^53^. For the purpose of this study, we intend to take a more pragmatic approach to the identification and selection of outcome definitions and measures. For all potential core outcomes (categories A and B) identified during the Delphi study, we will identify the definitions and outcome measures that were used in the studies included in our umbrella review. In the case of an outcome that was not identified as part of our umbrella review, we will perform targeted literature searches to identify relevant outcome measures. Targeted literature searches of MEDLINE and the COSMIN database will be used to identify studies investigating the quality of the outcome measures. Our research team and PPI panel will review the available evidence and provisionally prioritise the use of a single outcome measure after consideration and discussion of the following ^54^: 1) the frequency with which the outcome measure has been used in existing research; 2) the time and resources necessary to use the outcome measure; and 3) the available data on their measurement properties as outlined in the COSMIN recommendations (validity, reliability, responsiveness and interpretability) ^53^. Recommendations regarding the selection of outcome measures will be presented during the consensus meeting.

### Stage 5: consensus meeting

A virtual face-to-face meeting will take place with representatives from each stakeholder group to discuss, vote and agree upon the final COS and the definitions and methods to be utilised to assess these outcomes. Approximately 16 experts involved in the Delphi survey will be invited to take part in the consensus meeting. This panel will be purposively sampled to ensure that it includes representatives from each stakeholder group and from a range of geographic locations. The meeting will commence with a presentation outlining the preliminary COS and the ‘supplementary outcomes’ list. This will be followed by a timed discussion between panel members and a final vote. Similar to other COSs, the definition for consensus will be at least 70% of participants voting for the outcome to be included and a minimum of one patient representative voting for the outcome to be included in the COS ^35^. Any outcomes not meeting these criteria will remain on the ‘supplementary outcomes’ list. Relevant arguments for or against the inclusion of an outcome will be noted along with the vote counts. Finally, recommendations regarding definitions and outcome measures will be discussed. The consensus panel will be invited to provide feedback and discuss the recommendations before finalising the selection of a single outcome measure and definition, where applicable, for every included outcome. Reasoning for all decisions will be described narratively in the final published consensus statement.

### Dissemination and implementation strategy

A multi-modal approach to the dissemination of this COS will be employed. This COS will be developed and reported according to the Core Outcome Set-STAndards for Reporting (COS-STAR) guidelines ^55^. The final COS will be published in a peer-reviewed journal and will be shared through national and international conference presentations, and the appropriate media channels. In addition, this study has been registered with COMET and the final COS will be published on their website. The final COS will also be disseminated through relevant professional and patient organisations to inform healthcare professionals and the public.

### Ethics requirements

Ethics approval for this study has been granted by the Faculty of Education and Health Sciences Research Ethics Committee at the University of Limerick (EHSREC No: 2021_06_12). Participants in the Delphi survey will be provided with a study information leaflet as part of the invitation. At the beginning of round one of the online survey, participants will consent to take part in the study. Participants will be given the option to withdraw without explanation from this study at any time. Participants’ personal data will only be accessed by members of the research team and all survey responses will be confidential.

### Public and patient involvement

**Figure 2.**
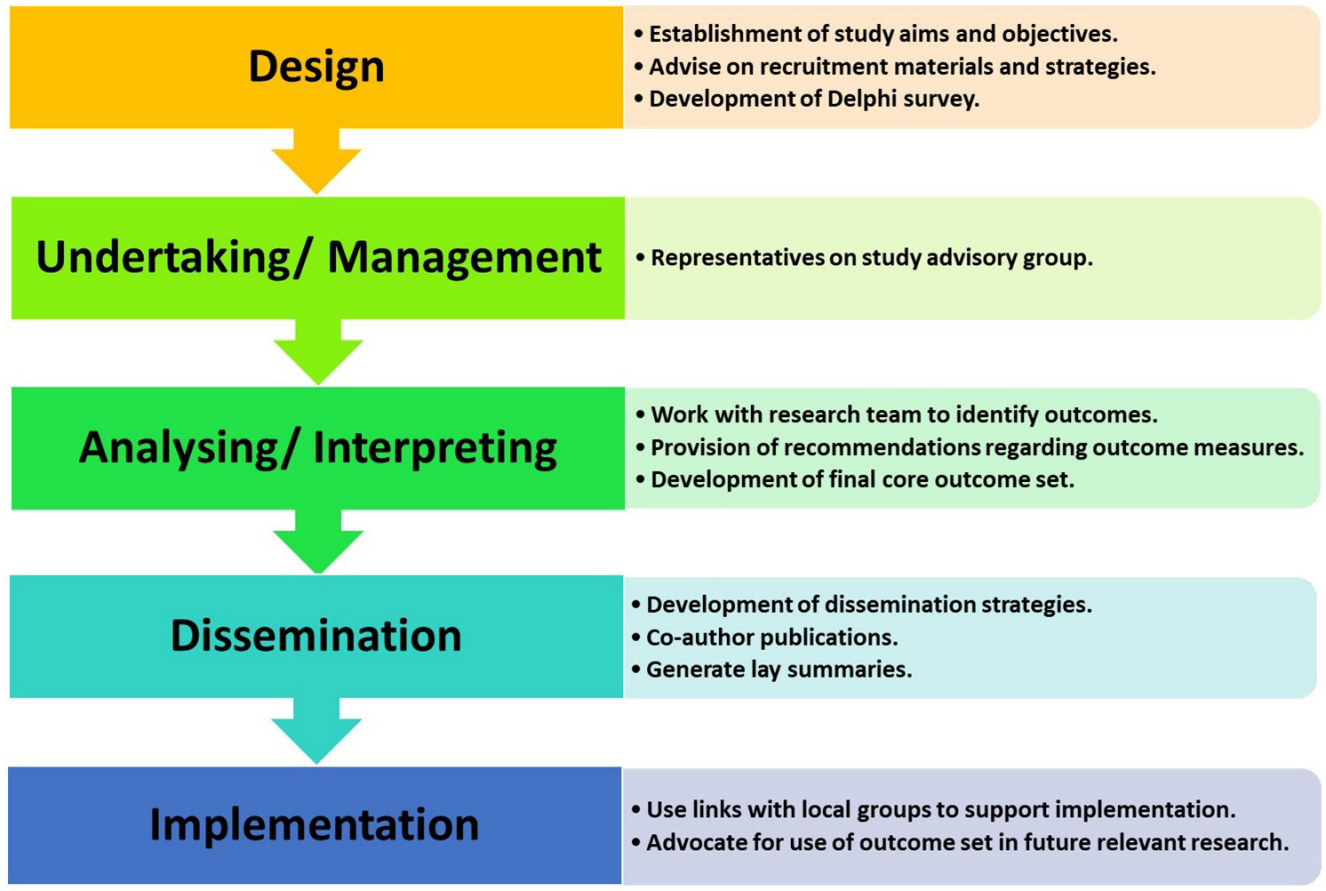
Overview of public and patient involvement in study.

A PPI panel has been established to guide the development of this COS. This PPI panel comprises of relevant stakeholders in Ireland, including individuals living with MS, PD and stroke, healthcare professionals, and representatives working with patient organisations. As outlined in Figure 2, this PPI panel will provide input and feedback from the design stage through to the dissemination and implementation stages of this study.

## Discussion

This protocol outlines the design of an international, multi-perspective Delphi consensus study to develop a COS for evaluating mixed-diagnosis falls prevention interventions for people with MS, PD and stroke. To our knowledge, the Delphi technique has not been previously used to gain consensus on a COS in this subject area. Given the high frequency of falls and their associated negative consequences among individuals with these neurological conditions, falls prevention is a priority for research and the provision of services. The establishment of an international standard for the assessment of outcomes would allow for transparent and coordinated falls research for people with these neurological conditions, facilitating advancements in this research field. The successful development and implementation of a COS would enable pooling of data, the conduction of meta-analyses and the cross-comparison of findings, aiding progress in the design and provision of effective evidence-based mixed-diagnosis falls prevention interventions for people with MS, PD and stroke. Once published, researchers investigating the effectiveness of falls prevention interventions for these conditions will have a well-founded rationale for the assessment of outcomes based on input from key stakeholders, thereby reducing heterogeneity and selective reporting of outcomes. Additionally, clinicians and service-planners/policy-makers will be better placed to compare research findings to guide clinical decision-making, optimising the translation and implementation of evidence-based falls prevention interventions into practice.

## Data availability

No data are associated with this article.

### Reporting guidelines

Figshare: COS-STAP Checklist for 'Protocol for the development of a core outcome set for evaluating mixed-diagnosis falls prevention interventions for people with Multiple Sclerosis, Parkinson’s Disease and stroke', https://doi.org/10.6084/m9.figshare.16669681.v1 ^37^.

Data are available under the terms of the Creative Commons Attribution 4.0 International license (CC-BY 4.0).
