# Supplementary material for: Protocol for the development of a core outcome set for evaluating mixed-diagnosis falls prevention interventions for people with Multiple Sclerosis, Parkinson’s Disease and stroke
Source: HRB Open Res. 2022 May 6;4:123. Originally published 2021 Nov 26. [Version 2] doi: 10.12688/hrbopenres.13459.2 (PMC9123332; doi:10.12688/hrbopenres.13459.2)
Supplement: Peer review history

# Peer review: Reviewer response for version 2

**DOI:** 10.21956/hrbopenres.14802.r31997

**Published:** 2022-05-20

**Authors:** Catriona McDaid, Rachel Cunningham-Burley

**Affiliations:** 1 Department of Health Sciences, York Trials Unit, University of York, York, UK; 2 University of York, York, UK

Thank-you to the authors for their detailed response, clarifications and amendments. We have no further comments.

Is the study design appropriate for the research question?

Yes

Is the rationale for, and objectives of, the study clearly described?

Yes

Are sufficient details of the methods provided to allow replication by others?

Partly

Are the datasets clearly presented in a useable and accessible format?

Not applicable

Reviewer Expertise:

applied health research, clinical trials, systematic reviews, musculoskeletal

We confirm that we have read this submission and believe that we have an appropriate level of expertise to confirm that it is of an acceptable scientific standard.

# Peer review: Reviewer response for version 2

**DOI:** 10.21956/hrbopenres.14802.r31998

**Published:** 2022-05-10

**Authors:** Michelle Cameron

**Affiliations:** 1 Department of Neurology, VA Portland Health Care System, Oregon Health & Science University, Portland, OR, USA

Thank you for addressing my concerns.

Is the study design appropriate for the research question?

Yes

Is the rationale for, and objectives of, the study clearly described?

Yes

Are sufficient details of the methods provided to allow replication by others?

Yes

Are the datasets clearly presented in a useable and accessible format?

Not applicable

Reviewer Expertise:

Falls and mobility in people with multiple sclerosis.

I confirm that I have read this submission and believe that I have an appropriate level of expertise to confirm that it is of an acceptable scientific standard.

# Peer review: Reviewer response for version 1

**DOI:** 10.21956/hrbopenres.14669.r31431

**Published:** 2022-02-28

**Authors:** Catriona McDaid, Rachel Cunningham-Burley

**Affiliations:** 1 University of York, York, UK; 2 Department of Health Sciences, York Trials Unit, University of York, York, UK

This clearly written paper provides an overview of the methods for the development of a core outcome set for use in the evaluation of falls prevention interventions applicable to mixed groups of people with stroke, Parkinson’s Disease and Multiple Sclerosis. A pragmatic rationale is presented that it is often not feasible to implement interventions for single condition groups and these conditions share similar risk factors. It could be clearer why these specific three conditions and not a wider range of conditions. Whilst you state that there are similarities in the falls risk in these three conditions, due to a number of common factors, it would be good to substantiate this further, particularly how these risks may differ, by virtue of the underlying neurological condition, from falls risk more generally. In addition, it is implied in the introduction that there is no COS for the individual conditions – this could be clearer and how this was established, for example through a search of COMET.

 The paper is scientifically sound and the methods are appropriate to answer the study objectives: the authors identify an explicit scope in terms of the population and proposed use of the COS, identify how the initial items for the Delphi will be identified (full details of the systematic review protocol are available separately), the process for undertaking the Delphi including the threshold for retention and removal of items and how they will identify relevant outcome measures. The methods mitigate against a single group dominating or unduly influencing the output from Delphi and consensus meeting. Ethical approval has been granted, though it could be clearer whether this extends to the qualitative study. The study is registered on COMET.

 Some specific suggestions for clarification in the protocol or for consideration when undertaking the study:

- It is a strength of the research that focus groups are planned to obtain the views of people with the three conditions of interest, as the authors correctly point out, the items derived from existing trials are more likely to reflect healthcare professional and researcher perspectives. However, more detail on the plans for the qualitative study would be helpful. How many focus groups are planned, what size will the groups be, what sampling methods will be used to capture a range of perspectives across conditions and within groups are captured? How will people be identified for inclusion in this part of the study, how will information be sought?
- Attrition can be very high from online surveys so aiming to recruit 15 from each group at the outset may be a bit risky to achieve the planned target of the suggested 10-18 per stakeholder group throughout the study. This only allows for one-third attrition. If feasible, consideration could be given to a larger initial pool. There are plans to achieve a geographic and gender balance. It could be clearer what is meant by geographic – urban-rural and/or across countries? Balance across conditions for patients and healthcare professionals is also likely to be important in order to be confident that the COS reflects the priorities of all three disease groups. However, no plans are described regarding this. You may also wish to consider the gender balance seen among the stakeholder groups; for example, MS is three times more common in females. Related to this it could be clearer in Stage 3 whether information about which condition patient participants have and duration of diagnosis or areas of clinical expertise (or generalist) for HCP participants will be gathered as part of the demographic data.
- Given the nature of the included neurological conditions, some participants may have communication or other difficulties that may make participation challenging.  Are there any plans to encourage the involvement of people who may require the support of a carer or family member to participate in the study either for health reasons, difficulties communicating or where they are not confident IT users? Will there be an option for an alternative means of completion of the rounds of surveys, other than electronically, to include those who may not have access to online technologies? Ideally, the perspectives of a wide range of patient groups would be captured. This also applies to the qualitative study.
- It is a strength that a third round will be used if necessary; however, it is not clear what would instigate the use of the third round.
- At the bottom of P5, it states that “All outcomes will be brought forward from round one to round two”. This suggests that the threshold criteria will not be applied at the end of round one – is this your intention or should the sentence refer to all newly elicited outcomes?
- It is a strength of the study that patients and the public will be involved at key stages of the study. However, given that the group also includes healthcare professionals ‘stakeholder group’ may be a more appropriate description than Patient and Public Involvement panel.

Is the study design appropriate for the research question?

Yes

Is the rationale for, and objectives of, the study clearly described?

Yes

Are sufficient details of the methods provided to allow replication by others?

Partly

Are the datasets clearly presented in a useable and accessible format?

Not applicable

Reviewer Expertise:

applied health research, clinical trials, systematic reviews, musculoskeletal

We confirm that we have read this submission and believe that we have an appropriate level of expertise to confirm that it is of an acceptable scientific standard, however we have significant reservations, as outlined above.

# Response

**Published:** 2022-05-03

**Authors:** Nicola O'Malley

**Affiliations:** University of Limerick, Ireland

Many thanks for your time spent reviewing this protocol, and for your constructive and insightful feedback and comments. We have reflected upon your feedback and revised our manuscript in line with it. Please see below a detailed point-by-point response to all comments (reviewer's comments in **bold** and authors’ responses in black font).

**Comment 2.1:** **This clearly written paper provides an overview of the methods for the development of a core outcome set for use in the evaluation of falls prevention interventions applicable to mixed groups of people with stroke, Parkinson’s Disease and Multiple Sclerosis. A pragmatic rationale is presented that it is often not feasible to implement interventions for single condition groups and these conditions share similar risk factors. It could be clearer why these specific three conditions and not a wider range of conditions. Whilst you state that there are similarities in the falls risk in these three conditions, due to a number of common factors, it would be good to substantiate this further, particularly how these risks may differ, by virtue of the underlying neurological condition, from falls risk more generally. In addition, it is implied in the introduction that there is no COS for the individual conditions – this could be clearer and how this was established, for example through a search of COMET.**

 As discussed in Response 1.2, the three most common neurological conditions presenting for falls prevention interventions are MS, PD and stroke. Additionally, we have evidence to demonstrate that falls risk factors and subsequent treatment approaches have many similarities across these three conditions. Consequently, we believe that these three conditions are the most appropriate for commencing this mixed-diagnosis approach to falls prevention interventions to facilitate implementation. We have now updated our introduction to clarify the rationale for including the three neurological conditions. Additionally, we have clarified in the introduction that a search of COMET was undertaken to establish whether a COS exists.

**Comment 2.2:** **The paper is scientifically sound and the methods are appropriate to answer the study objectives: the authors identify an explicit scope in terms of the population and proposed use of the COS, identify how the initial items for the Delphi will be identified (full details of the systematic review protocol are available separately), the process for undertaking the Delphi including the threshold for retention and removal of items and how they will identify relevant outcome measures. The methods mitigate against a single group dominating or unduly influencing the output from Delphi and consensus meeting. Ethical approval has been granted, though it could be clearer whether this extends to the qualitative study. The study is registered on COMET.**

 Ethics has been granted by the Faculty of Education and Health Sciences Research Ethics Committee at the University of Limerick for both the qualitative study and the Delphi consensus study. This has been clarified under ‘Ethics requirements’.

**Comment 2.3:** **It is a strength of the research that focus groups are planned to obtain the views of people with the three conditions of interest, as the authors correctly point out, the items derived from existing trials are more likely to reflect healthcare professional and researcher perspectives. However, more detail on the plans for the qualitative study would be helpful. How many focus groups are planned, what size will the groups be, what sampling methods will be used to capture a range of perspectives across conditions and within groups are captured? How will people be identified for inclusion in this part of the study, how will information be sought?**

 Thank you for this suggestion. Given the recent criticisms of pre-empting a data saturation point in advance to justify sample size, we will not have a pre-defined sample size but will rather make a decision regarding sample size during peer debrief sessions based on the adequacy of the collected data to answer the research question. Recruitment will adhere to the principles of purposeful maximum variation and snowball sampling to ensure we are capturing a wide range of perspectives. Participants will be recruited through support groups and community services. The methods for our qualitative study have been clarified further in the paragraph ‘Focus groups with people living with MS, PD and stroke’.

**Comment 2.4:** **Attrition can be very high from online surveys so aiming to recruit 15 from each group at the outset may be a bit risky to achieve the planned target of the suggested 10-18 per stakeholder group throughout the study. This only allows for one-third attrition. If feasible, consideration could be given to a larger initial pool. There are plans to achieve a geographic and gender balance. It could be clearer what is meant by geographic – urban-rural and/or across countries? Balance across conditions for patients and healthcare professionals is also likely to be important in order to be confident that the COS reflects the priorities of all three disease groups. However, no plans are described regarding this. You may also wish to consider the gender balance seen among the stakeholder groups; for example, MS is three times more common in females. Related to this it could be clearer in Stage 3 whether information about which condition patient participants have and duration of diagnosis or areas of clinical expertise (or generalist) for HCP participants will be gathered as part of the demographic data.**

 Thank you for this suggestion. We will aim to recruit 20 people per stakeholder group to retain a minimum sample of 10 per stakeholder group, with equal representation of all three conditions in the patient group. Geographic balance refers to countries rather than urban/rural. We anticipate recruiting more females with MS and males with PD, in line with gender distribution for those conditions and therefore anticipate a gender-balanced sample overall. If an imbalance occurs, we can utilise our snowball sampling methods to recruit additional people. Apologies for the lack of clarification. The above detail has now all been outlined in the ‘Participants’ section.

 Specific demographic data that will be requested from the patient stakeholder group include neurological diagnosis, time since diagnosis, falls history and mobility status. Clinicians will be asked to provide their qualifications, which diagnostic groups they work with, how long they have been working with those groups, and what percentage of their caseload they account for. This has now been clarified under ‘Delphi survey – round one’.

**Comment 2.5:** **Given the nature of the included neurological conditions, some participants may have communication or other difficulties that may make participation challenging.  Are there any plans to encourage the involvement of people who may require the support of a carer or family member to participate in the study either for health reasons, difficulties communicating or where they are not confident IT users? Will there be an option for an alternative means of completion of the rounds of surveys, other than electronically, to include those who may not have access to online technologies? Ideally, the perspectives of a wide range of patient groups would be captured. This also applies to the qualitative study.**

 As mentioned in the methods section, our stakeholder group will be involved in the design of the survey to make it as user-friendly as possible (for example allowing participants to complete the survey across several sessions rather than in one sitting). In addition to this, we will make provisions as appropriate to facilitate participation in both the qualitative and consensus studies. Specific details regarding these provisions for the qualitative study have been added to the section ‘Focus groups with people living with MS, PD and stroke’, and for the consensus study under ‘Stage 2: development of the Delphi survey’.

**Comment 2.6:** **It is a strength that a third round will be used if necessary; however, it is not clear what would instigate the use of the third round.**

 If there are outcomes near the thresholds for consensus (rated 7-9 by more than 50% of participants and rated 1-3 by less than 15% of participants), then a third round of the Delphi survey will be completed. The survey will be terminated following a third round regardless of whether or not all outcomes have achieved consensus. This additional information has been outlined under the section ‘Delphi survey – round three (if required).

**Comment 2.7:** **At the bottom of P5, it states that “All outcomes will be brought forward from round one to round two”. This suggests that the threshold criteria will not be applied at the end of round one – is this your intention or should the sentence refer to all newly elicited outcomes?**

 Yes, it is our intention to bring all outcomes forward to round two. A key feature of the Delphi method is the provision of controlled feedback to participants, For that reason, we will not be applying the consensus threshold criteria at the end of round one but instead will be bringing forward all outcomes to round two to allow participants to reconsider their own responses. This has been clarified in the manuscript under ‘Delphi survey – round one’.

****

**Comment 2.8:** **It is a strength of the study that patients and the public will be involved at key stages of the study. However, given that the group also includes healthcare professionals ‘stakeholder group’ may be a more appropriate description than Patient and Public Involvement panel.**

 Thank you for this suggestion. The phrase ‘Patient and Public Involvement panel’ has been updated to ‘stakeholder group’ accordingly throughout this paper.

# Peer review: Reviewer response for version 1

**DOI:** 10.21956/hrbopenres.14669.r30951

**Published:** 2021-12-06

**Authors:** Michelle Cameron

**Affiliations:** 1 Department of Neurology, VA Portland Health Care System, Oregon Health & Science University, Portland, OR, USA

This manuscript describes a protocol for developing a core outcome set (COS) for fall prevention interventions in people with multiple sclerosis (MS), Parkinson's Disease (PD), and stroke. The manuscript is well written. The language is clear. The approach is robust, with the appropriate stakeholders and iterative sequence. Thus, based on your assigned review questions, this manuscript meets the criteria of having clearly described rationale and objectives, having an appropriate study design for the research question and, providing sufficient detail to allow for replication.

 However, I do have one substantial concern. I question if the research question being asked is the ideal one. Why have the authors limited the COS to people with 3 specific neurological conditions when falls are more ubiquitous? I would think a better COS for mixed-diagnosis falls prevention interventions would be diagnosis agnostic and include anyone who either has fallen often or is thought to be at high fall risk. Why would you want different outcomes related to falls in those with MS, PD and stroke compared to older adults with often multiple comorbidities contributing to their fall risk? I, therefore, suggest that the authors either broaden their target population or give a clear rationale for why these specific 3 diagnoses and no others are included.

Is the study design appropriate for the research question?

Yes

Is the rationale for, and objectives of, the study clearly described?

Yes

Are sufficient details of the methods provided to allow replication by others?

Yes

Are the datasets clearly presented in a useable and accessible format?

Not applicable

Reviewer Expertise:

Falls and mobility in people with multiple sclerosis.

I confirm that I have read this submission and believe that I have an appropriate level of expertise to confirm that it is of an acceptable scientific standard, however I have significant reservations, as outlined above.

# Response

**Published:** 2022-05-03

**Authors:** Nicola O'Malley

**Affiliations:** University of Limerick, Ireland

Many thanks for your time spent reviewing this protocol, and for your constructive and insightful feedback and comments. We have reflected upon your feedback and revised our manuscript in line with it. Please see below a detailed point-by-point response to all comments (reviewer's comments in **bold** and authors’ responses in black font).

**Comment 1.1: This manuscript describes a protocol for developing a core outcome set (COS) for fall prevention interventions in people with multiple sclerosis (MS), Parkinson's Disease (PD), and stroke. The manuscript is well written. The language is clear. The approach is robust, with the appropriate stakeholders and iterative sequence. Thus, based on your assigned review questions, this manuscript meets the criteria of having clearly described rationale and objectives, having an appropriate study design for the research question and, providing sufficient detail to allow for replication.**

 Thank you for this positive feedback.

**Comment 1.2:** **However, I do have one substantial concern. I question if the research question being asked is the ideal one. Why have the authors limited the COS to people with 3 specific neurological conditions when falls are more ubiquitous? I would think a better COS for mixed-diagnosis falls prevention interventions would be diagnosis agnostic and include anyone who either has fallen often or is thought to be at high fall risk. Why would you want different outcomes related to falls in those with MS, PD and stroke compared to older adults with often multiple comorbidities contributing to their fall risk? I, therefore, suggest that the authors either broaden their target population or give a clear rationale for why these specific 3 diagnoses and no others are included.**

 Thank you for this thought-provoking comment. As you are aware, there is an existing core outcome set for fall-injury prevention interventions among older adults. Consequently, falls research for older adults can be pooled, with many systematic reviews and meta-analyses providing a solid evidence-base to support falls prevention programmes in older adults. Many people with neurological conditions are younger and therefore do not fit under that umbrella despite having high falls rates. Consequently, the focus of this core outcome set will not be on older adults to facilitate progress in falls research among other populations that are not as well researched.

 Health services globally, and in Ireland, are predominantly structured around diagnostic categories/models of care, one of which is neurology. Therefore, to align with the implementation strategies within these health services, this proposed core outcome set will focus solely on falls prevention interventions for people with neurological conditions, namely MS, PD and stroke. We are proposing the design and evaluation of mixed-diagnosis interventions for people with these neurological conditions as a pragmatic approach to overcome the challenges associated with delivering single-diagnosis interventions in the community. People who have been diagnosed with stroke, PD and MS have higher rates of falls than older adults and these are the three most common conditions presenting to physiotherapists in a rehabilitation setting. We have evidence to show that many falls risk factors and subsequent intervention approaches are similar across these three conditions. As such, we believe that the combination/selection of these three conditions is the most appropriate for commencing this mixed-diagnosis approach. Our introduction has been edited to clarify our intentions in relation to the selection of the three neurological conditions.
